# New Appliances and Things Medical

**Published:** 1911-01-21

**Authors:** 


### NEW APPLIANCES & THINGS MEDICAL.

[We shall be glad to receive at our Office, 28 & 29 Southampton Street, Strand, London, W.C., from the manufacturers, specimens of all new preparations and appliances.]

### NUVITE.

(THE NUVITÉ CO., LTD., 166 UNTHANK ROAD, NORWICH.)

WE have received a bottle of the above restorative wine, which we understand from the accompanying literature is port wine with which has been incorporated elements similar to those found in the tissues in a pre-digested form. The literature also states that no drug, such, for instance, as cocaine, is contained in Nuvité. It seems therefore to be good port wine which has had its nutritive value increased by the addition of organic nutritive substances. An abundance of literature is supplied testifying to its physiological effect as a restorative. The dose is one wineglassful after meals, and one or two dessertspoonfuls for children. It is pleasant tasting and makes a very palatable after-meal draught.

### HARVEY'S SUGAR-FREE PALE ALE.

(SOLE WEST END AGENTS, BONTHRON & CO., 106 REGENT STREET, W.)

MANY of our readers will no doubt remember that in the report of THE HOSPITAL Beer Commission we discussed at some length the chemical composition of good beer, especially so far as concerned its non-alcoholic constituents. The most important of these are maltose and dextrine. The presence of these substances, valuable as they are to the ordinary consumer, renders beer, speaking generally, an unsuitable beverage in diabetes and in some cases of the gouty diathesis. To many patients the bitter properties and the alcohol in beer are useful quite apart from the above substances. In the beer before us the carbohydrates have been reduced to a minimum, and hence the beverage is quite suitable for the use of the diabetic and gouty. We think the preparation is distinctly to be recommended.

### THE IDEAL FLOOR-POLISH.

(RONUK, LTD., Portslade, near Brighton; London Agency, 86 York Road, Lambeth, S.E.)

To many institutional workers "Ronuk," like good wine, needs no bush. But it is possible that there are some superintendents who are not aware yet of the admirable merits of this firm's floor-polish. Standard Ronuk is a clean, effective, convenient, antiseptic preparation, easy to apply, free from excessive grease or smell, not liable to render a hardwood floor excessively slippery, and withal cheap and always ready when wanted. It is now used in so many first-class hospitals that it is almost a matter for surprise when one visits a hospital and finds that its floors are not polished with Ronuk. The small cottage hospital especially should make a point of trying the preparation. The company undertakes to keep institution floors in good order on contract, and several hospitals in London and elsewhere have already availed themselves of the advantageous terms offered to secure the services of the highly skilled floor-polishers trained by the firm. We hope in a future issue to deal at length with the whole matter of floor polishing and to review the good work that has been done by Ronuk, work that should be known to all hospital workers. In addition to the floor-polish Ronuk supplies metal and boot polishes, household sanitary cleansers, and ballroom floor-powders, all of a very fine quality and fully deserving attention. The prices charged are throughout reasonable, and the specialities made by this firm can therefore be thoroughly recommended.

